# Me and my migraine: impact survey for children and young people

**DOI:** 10.1186/1129-2377-14-S1-P31

**Published:** 2013-02-21

**Authors:** D Burn, R Markham, S Lipscombe, I Abu-Arafeh

**Affiliations:** 1 Migraine Association, UK; 2 Royal Sussex Hospital, UK; 3 Forth valley Royal Hospital, UK

## Objective

This study aims to assess the impact of migraine in children and young people. Population: A group of children with migraine identified from 3 sources: (a) children with migraine who were identified through a random sample of schoolchildren, (b) children with migraine attending specialist clinics and (c) children with migraine who are members related to members of Migraine Action. A control group of children were selected at random from schoolchildren.

## Methods

The impact of migraine on children education, school attendance, social life and relationships was assessed by filling a special questionnaire designed for this purpose by the two groups of children. Data were stored and analysed on Excel.

## Results

Questionnaires were fully completed by 506 children (257 had migraine and 249 controls). Twenty three questionnaires were partially completed and were excluded.70% were females and age range was 1-18 years (83% between 10-16 years). Children with migraine missed on average 9.7 days of school due to illness over the past 3 months compared to 2.7 days in children without migraine. 85% of children with migraine were prevented from taking part in activities and hobbies on at least one occasion, 60% missed at least one special family event and 80% had to cancel plans with friends at least once. Over 50% of children with migraine felt less confident, felt it was hard to carry out homework or chores and felt that migraine stopped them doing well at school. Conclusions The impact of migraine on children and adolescents is often underestimated and a better management of childhood migraine may help improve children’s quality of life.

